# 3D-printed patient-specific instrumentation decreases the variability of patellar height in total knee arthroplasty

**DOI:** 10.3389/fsurg.2022.954517

**Published:** 2023-01-10

**Authors:** Junfeng Wang, Xiaohua Wang, Bin Sun, Liang Yuan, Ke Zhang, Bin Yang

**Affiliations:** Department of Orthopedics, Peking University International Hospital, Beijing, China

**Keywords:** total knee arthroplasty, patient-specific instrumentation, anterior condylar offset, posterior condylar offset, posterior tibial slope

## Abstract

**Objective:**

Three-dimensionally (3D) printed patient-specific instrumentation (PSI) might help in this regard with individual design and more accurate osteotomy, but whether the utility of such instrumentations minimizes the variability of patellar height in total knee arthroplasty (TKA) and the reasons for this effect are unknown. Our aim is to compare and analyze the variability of patellar height with PSI and conventional instrumentation (CI) in TKA.

**Methods:**

Between March 2018 and November 2021, 215 patients with severe knee osteoarthritis who were treated with primary unilateral TKA were identified for this observational study. The patients were divided into the CI-TKA group and PSI-TKA group according to the osteotomy tools used in TKA. Preoperative and postoperative radiographic parameters including hip–knee–ankle angle (HKA), posterior tibial slope (PTS), Insall–Salvati ratio, modified Caton–Deschamps (mCD) ratio, anterior condylar offset (ACO), and posterior condylar offset (PCO) were evaluated.

**Results:**

The groups were similar in patients' demographic data, clinical scores, and radiographic parameters preoperatively. Overall, according to the results of the Insall–Salvati ratio, postoperative patellar height reduction was noted in 140 patients (65.1%). Interestingly, the variability of patellar height was smaller in the PSI-TKA group. Radiographic evaluation revealed that the Insall–Salvati ratio after TKA had a minor change in the PSI-TKA group (*p* = 0.005). Similarly, the mCD ratio after TKA also had a minor change in the PSI-TKA group (*p* < 0.001). Compared to those in the CI-TKA group, the ACO (*p* < 0.001) and PCO (*p* = 0.011) after TKA had a minor change in the PSI-TKA group, but no minor PTS change (*p* = 0.951) was achieved in the PSI-TKA group after TKA. However, even with 3D-printed patient-specific instrumentation, there were still significant reductions in patellar height, ACO, PCO, and PTS after TKA (*p* < 0.001).

**Conclusion:**

The variability of patellar height was sufficiently minimized with more accurate anterior and posterior femoral condyle osteotomy when 3D printed PSI was used. Furthermore, there was a trend in over-resection of the femoral anterior and posterior condyle and a marked reduction in PTS during TKA, which could lead to a change in patellar height and might result in more patellofemoral complications following TKA.

**Level of evidence:**

Level II.

## Introduction

The patella is an essential component of the biomechanics of the knee joint; optimal patellar position improves quadriceps efficiency by increasing the moment arm of the knee extensor mechanism (1). Patella baja or alta can cause an alteration in the normal biomechanics of the knee joint and can give rise to anterior knee pain, limited range of movement (ROM), knee extensor lag, and reduced functional scores (2–4).

Total knee arthroplasty (TKA) is a relatively common operation. Although beneficial to the well-being of the patient, it may cause biomechanical changes and complications in the patellofemoral joint (5). During TKA, the patellar height may be changed due to abnormal femoral or tibial cuts made during the operation, excessive soft tissue release, abnormal placement of prosthetic components, and excessive resection of the infrapatellar fat pad (5, 6). Therefore, minimizing the variation of patellar height after TKA is crucial for better postoperative satisfaction and fewer patellofemoral joint complications.

Computer tomography (CT)-based three-dimensionally (3D) printed patient-specific instrumentation (PSI) is an innovative alternative to conventional instrumentation (CI) for TKA (7). Based on the reconstructed 3D images, surgeons can determine the size and position of the implant, which is helpful for an individual design according to patient's anatomical characteristics in the femur and tibia, to adapt to the patient's special anatomical shape and variation and increase the accuracy of osteotomy (8–11). These results suggest that there might be some clinical value of 3D printed patient-specific instrumentation for reducing the difference between the prosthesis position and patient's preoperative anatomy in TKA. However, to our best knowledge, there are no studies on the additional value of 3D printed patient-specific instrumentation for decreasing the variability of patellar height in TKA. Furthermore, to our knowledge, there are no studies evaluating and analyzing the reasons for reducing the variability of patellar height for 3D printed patient-specific instrumentation.

While comparing 3D printed patient-specific instrumentation with conventional instrumentation in TKA, we aimed to answer three main study questions: (1) Does the overall patellar height change after TKA? (2) Does the variability of patellar height vary between 3D printed patient-specific instrumentation and conventional instrumentation in TKA? (3) Does the reduction in the variability of patellar height stem from restraining the variation in the anterior condylar offset (ACO), posterior condylar offset (PCO), and posterior tibial slope (PTS) with 3D printed patient-specific instrumentation in TKA?

## Patients and methods

### Study design

A case–control study design was utilized. From March 2018 to November 2021, 215 patients diagnosed with severe knee osteoarthritis who were treated with primary unilateral TKA were identified for this observational study. Patients with inflammatory arthritis, post-traumatic arthritis, knee valgus deformities, severe extra-articular deformities, or poor imaging quality were excluded. There were 27 men and 188 women. The mean age of patients was 67.7 ± 6.0 years (range 51–81 years), and the mean body mass index (BMI) was 27.2 ± 3.8 kg/m^2^ (range 18.0–38.0 kg/m^2^). All patients received and accepted informed consent to participate in this study, which was approved by the medical ethics committee of Peking University International Hospital [2020-036(BMR)].

This was a real-world study, and the surgical grouping was at the discretion of the patient. The patients were divided into the conventional instrumentation (CI) group and the patient-specific instrumentation (PSI) group according to the osteotomy tools used in TKA. The CI-TKA group was performed using a GII fixed-bearing PS total knee system (Smith & Nephew, USA), and PSI-TKA group was performed using an A3 fixed-bearing PS total knee system (AK Medical, China).

Preoperative clinical variables including age, sex, affected side, body mass index, range of motion (ROM), Knee Society score (KSS), Knee Society function score (KSFS) (12), and Western Ontario and McMaster Universities Osteoarthritis Index (WOMAC) (13) were recorded.

### Radiographic assessment

**Figure 1:**
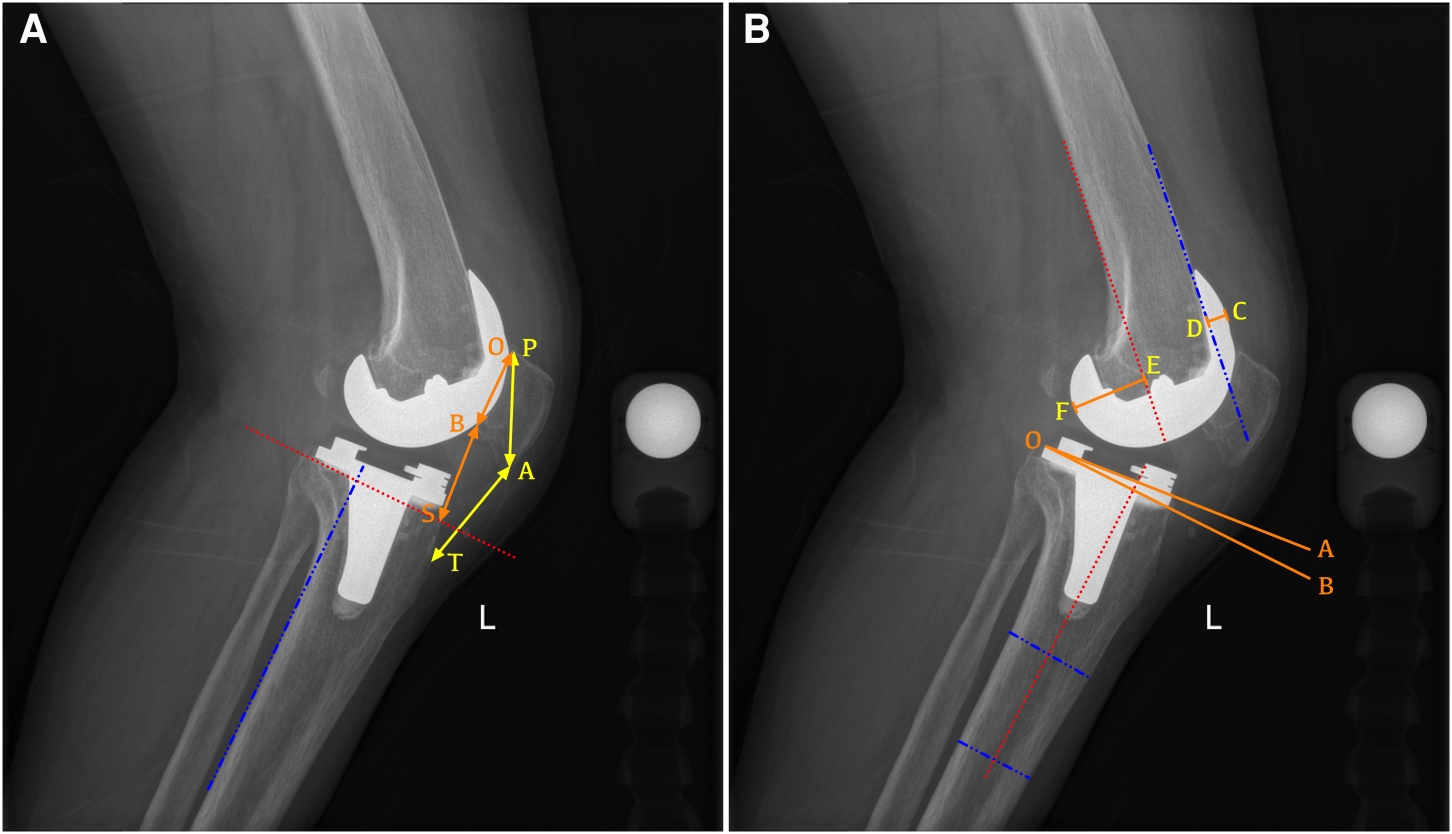
Radiographic measurement of the lateral view after PS-TKA. (**A**) Example of a radiograph demonstrating how to measure the Insall–Salvati ratio (AT/AP) and modified Caton–Deschamps ratio (BS/BO). AT, length of the patellar tendon; AP, longest diagonal length of the patella; B0, length of the articular surface of the patella; BS, distance from the distal portion of the articular surface of the patella (B) to the intersection between the line perpendicular to the tibial posterior cortex elevated at the tip of the fibular head and the tibial anterior cortex (S). (**B**) Example of a radiograph demonstrating how to measure the proximal anatomic axis posterior tibial slope (the angle between lines AO and BO), the anterior condylar offset (CD, the distance between the anterior femoral cortical margin and the anterior margin of the femoral condyles), and posterior condylar offset (EF, the distance between the posterior femoral cortical margin and the posterior margin of the femoral condyles).

All patients underwent radiographic assessment before and after surgery, and the radiographic technique was performed following standardized procedures (14). A full-length weight-bearing anteroposterior radiograph of the lower extremity was taken in full extension with the patient standing, the leg extended, and the knee ensured not to be rotated. For lateral radiographs, the medial and lateral femoral condyles were overlapped at approximately 30° of knee flexion to indicate an adequately rotated radiograph. The variables were all measured by two orthopedic surgeons who were blinded to patients' information using a PACS system (Centricity; General Electric, USA), and the average of the two measurements was used for the analysis. Preoperative and postoperative radiographic parameters including hip–knee–ankle angle (HKA), posterior tibial slope (PTS) (15), Insall–Salvati ratio (16), modified Caton–Deschamps (mCD) ratio (17), anterior condylar offset (ACO), and posterior condylar offset (PCO) (18) were evaluated according to the method in the previous literature (Figure 1).

### 3D printed patient-specific instrumentation

**Figure 2:**
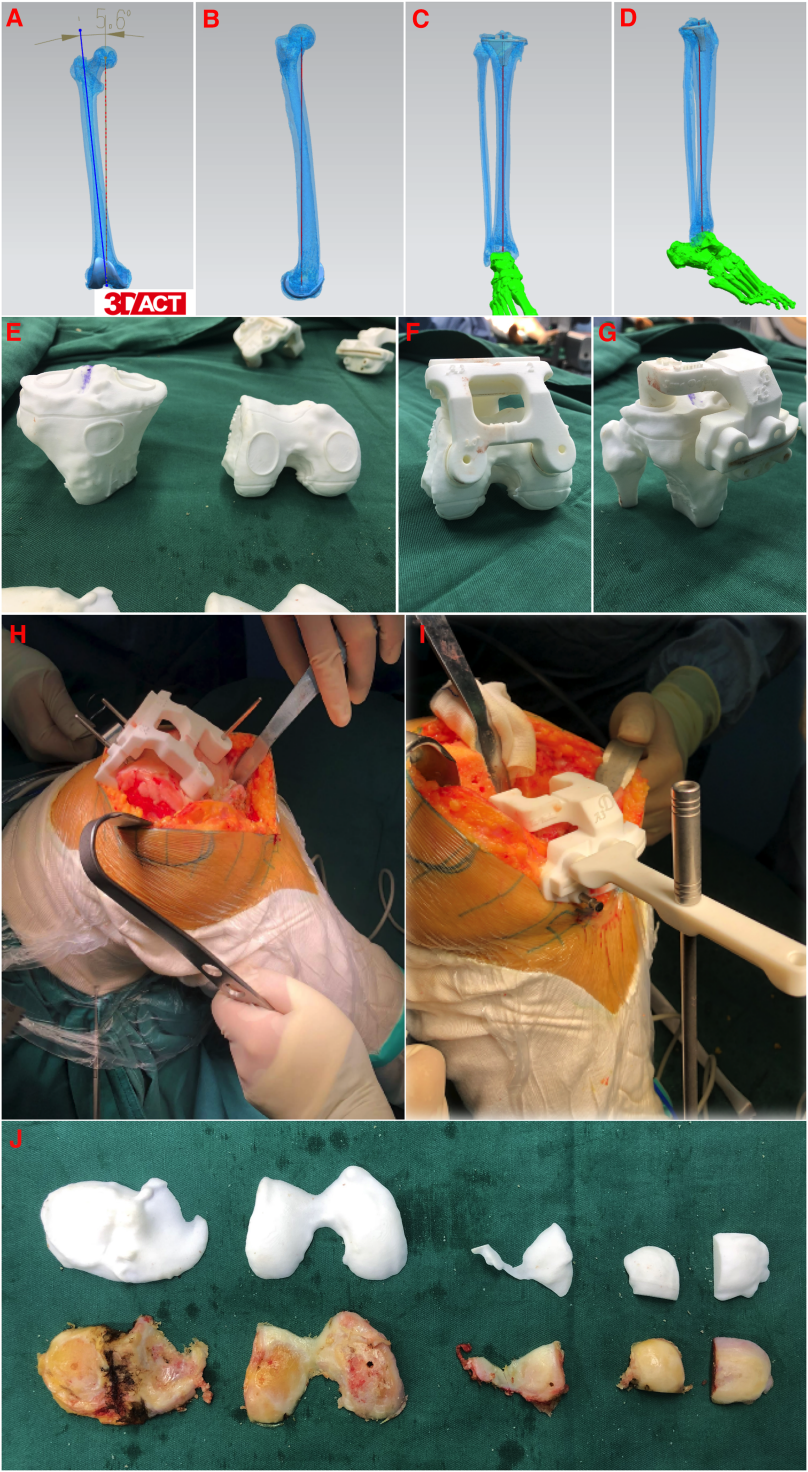
Design and intraoperative application of 3D printed patient-specific instrumentations. (**A–D**) CT-based preoperative planning of femoral and tibial prosthetic implant for anatomic reconstruction. (**E**) Reconstructed models of the patient's distal femur and proximal tibia, including markers for the location of the intraoperative osteotomy. (**F,G**) Confirming the good fit of 3D printed patient-specific instrumentations. (**H,I**) Intraoperative positioning and application of 3D printed patient-specific instrumentations. (**J**) Real-time comparison of preoperative designed osteotomy volume and actual osteotomy volume.

In the PSI-TKA group, prior to surgery, all enrolled patients underwent full-length lower extremity thin-slice CT scanning (SOMATOM Definition AS128, Siemens, Germany) on the affected side, with the scan thickness ≤1 mm. During CT scanning, the affected side leg was in a neutral position, and the patella was upward. The scanning range was from the femoral head to the ankle. Then, the CT scanning data were imported into Mimics 20.0 (Materialise, Belgium) in the DICOM format to reconstruct the model of the femur, tibia, and lower extremity alignment. All 3D models were exported as standard tessellation language files into Unigraphics NX software (Siemens, Germany). The alignment was measured based on anatomic landmarks, and the simulated osteotomy was performed in this software. The osteotomy surfaces of the distal femoral and proximal tibia were designed to be perpendicular to the mechanical axis of the lower extremity, and the coronal osteotomy surfaces of the femoral were designed to be parallel to the anatomical transepicondylar axis. During simulated osteotomy, the medical–engineering interaction was carried out according to patient's individual anatomical characteristics and pathological changes, and the parameters such as the osteotomy volume, prosthesis size, and prosthesis position were repeatedly adjusted until the final prosthesis adaptation was completed. After that, the intraoperative PSI osteotomy guide was printed with an industrial-grade 3D printer (EOS P110, Germany) according to the final preoperative design (Figures 2A–G). The printed material for PSI in this study was biocompatible polyamide (PA12) (19).

### Surgical procedures

All procedures were performed by one group of experienced surgeons using a fixed-bearing PS total knee system. Patients had combined spinal–epidural anesthesia unless contraindicated by a medical issue. Tourniquet was used for the entire procedure. The incision was adopted with a medial parapatellar approach, the patella was not inverted during the operation, the infrapatellar fat pad was minimally removed, and the patella replacement was not performed. The postoperative analgesia regimens were a cocktail of periarticular injection combined with femoral nerve block analgesia, and the rehabilitation training was completed by the same rehabilitation team.

In the CI-TKA group, distal femoral resection was performed using the intramedullary technique according to the femoral valgus angle. Femoral rotation was determined by the posterior femoral condylar axis with a 3° external rotation combined with double-checking with Whiteside's line and patient's soft tissue balance. The proximal tibial resection was performed by the extramedullary technique. The tibial component with an asymmetric tibial tray was rotated by the functional alignment method. After osteotomy, flexion and extension gap balancing was performed using spacer blocks to obtain equally symmetric gaps.

In the PSI-TKA group, after the femoral condyle was exposed, a custom curette was used to remove the cartilage on the anterior surface of the femoral condyle at the positioning point of the osteotomy guide so that the osteotomy guide was close to the subchondral bone. When the osteotomy guide was confirmed to be in good fit with the femoral condyle, positioning nails were implanted through the holes and then completed the distal femur osteotomy. The positioning nails in the distal femur were parallel to the anatomical transepicondylar axis (Figure 2H). The external rotatory osteotomy of the distal femur with the 5-in-1 metal cutting block was fixed according to the positioning of nails previously implanted on the distal femur. Then, the cartilage at the positioning point of the tibial plateau was removed, and the osteotomy guide was placed close to the subchondral bone to complete the ﻿proximal tibia osteotomy (Figures 2I,J). The remaining procedures were then carried out like the CI-TKA group.

### Statistical analysis

All data were analyzed using GraphPad Prism (version 9; GraphPad Software, USA). Continuous data were presented as a mean ± standard deviation. The Shapiro–Wilk test was used to test continuous variables for normal distribution. For continuous variables with a normal distribution, the comparison between groups was conducted using independent samples *t*-test or paired samples *t*-test; for continuous variables with non-normal distribution, the comparison between groups was performed using the Mann–Whitney *U* test. Categorical variables were compared between groups using Fisher's exact test. The values were considered significant when the two-tailed *p*-value of the difference was less than 0.05.

## Results

### General features of patients preoperatively

A total of 215 patients were enrolled in this study, including 76 patients (35.3%) in the CI-TKA group and 139 patients (64.7%) in the PSI-TKA group. Patients' demographics and clinical scores preoperatively are summarized in Table 1. Preoperative radiological data are summarized in Table 2. The patients' demographic data, clinical scores, and radiographic parameters preoperatively revealed no statistically significant differences between the two groups (Tables 1, 2), suggesting that the two groups were similar without significant differences in the baseline characteristics.

**Table 1:** Patients’ demographics and clinical scores preoperatively.

Variable	CI-TKA group (*n *= 76)	PSI-TKA group (*n *= 139)	*p* value
Age (years)	67.3 ± 6.1	68.0 ± 6.0	0.396
Gender (male/female)	10/66	17/122	0.844
Affected side (left/right)	36/40	68/71	0.828
BMI (kg/m^2^)	27.4 ± 3.8	27.1 ± 3.8	0.718
Preoperative ROM (°)	99.2 ± 23.8	99.5 ± 22.3	0.841
Preoperative KSS	51.4 ± 22.0	52.2 ± 19.4	0.796
Preoperative KSFS	52.4 ± 17.5	52.4 ± 18.4	0.824
Preoperative WOMAC	45.8 ± 20.0	48.6 ± 18.4	0.302

CI-TKA, total knee arthroplasty with traditional instruments; PSI-TKA, total knee arthroplasty with patient-specific instrumentation; BMI, body mass index; ROM, range of motion; KSS, American Knee Society knee score; KSFS, American Knee Society function score; WOMAC, Western Ontario and McMaster Universities Osteoarthritis Index.

**Table 2:** Preoperative radiographic parameters.

Parameters	CI-TKA group (*n *= 76)	PSI-TKA group (*n *= 139)	*p* value
Preoperative HKA angle (°)	168.4 ± 5.5	169.0 ± 5.1	0.539
Preoperative PTS (°)	11.0 ± 4.4	10.9 ± 3.6	0.816
Preoperative ACO (mm)	8.2 ± 2.0	8.1 ± 2.0	0.611
Preoperative PCO (mm)	33.2 ± 5.1	32.7 ± 4.0	0.406
Preoperative Insall–Salvati ratio	1.1 ± 0.1	1.1 ± 0.1	0.955
Preoperative mCD ratio	1.4 ± 0.1	1.4 ± 0.1	0.957

CI-TKA, total knee arthroplasty with traditional instruments; PSI-TKA, total knee arthroplasty with patient-specific instrumentation; HKA, hip–knee–ankle angle; PTS, posterior tibial slope; ACO, anterior condylar offset; PCO, posterior condylar offset; mCD, modified Caton–Deschamps.

### Variability of patellar height after TKA

**Figure 3:**
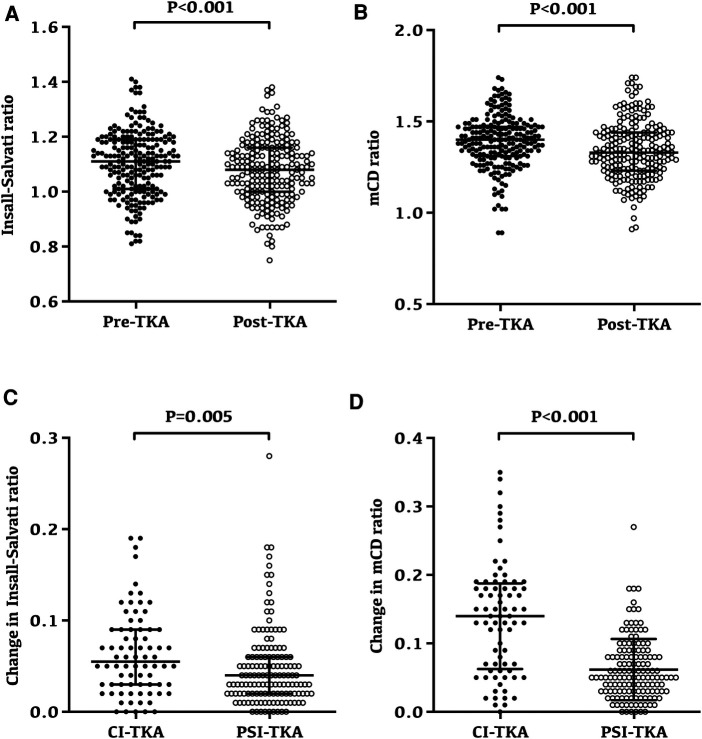
Changes and variability of patellar height following TKA. (**A**) Preoperative and postoperative Insall–Salvati ratio for all patients; the Insall–Salvati ratio was 1.11 ± 0.12 preoperatively and reduced to 1.08 ± 0.12 after TKA (*p* < 0.001). (**B**) Preoperative and postoperative modified Caton–Deschamps ratio for all patients; the modified Caton–Deschamps ratio was 1.39 ± 0.14 preoperatively and reduced to 1.34 ± 0.16 after TKA (*p* < 0.001). (**C**) Insall–Salvati ratio after TKA had a minor change in the PSI-TKA group (*p* = 0.005). (**D**) Modified Caton–Deschamps ratio after TKA also had a minor change in the PSI-TKA group (*p* < 0.001).

Overall, the patellar height after TKA was significantly lower than the preoperative patellar height (Figures 3A,B). According to the results of the Insall–Salvati ratio, the postoperative patellar height reduction was noted in 140 patients (65.1%). Among them, 87 patients (62.6%) in the PSI-TKA group and 53 patients (69.7%) in the CI-TKA group showed postoperative patellar height reduction. There was no statistical difference in the composition ratio of patellar height reduction between the two groups (*p* = 0.369). The Insall–Salvati ratio was 1.11 ± 0.12 preoperatively and reduced to 1.08 ± 0.12 after TKA (*p* < 0.001, Figure 3A). Similarly, the mCD ratio was 1.39 ± 0.14 preoperatively and reduced to 1.34 ± 0.16 after TKA (*p* < 0.001, Figure 3B).

Interestingly, the variability of patellar height in the PSI-TKA group was smaller (Figures 3C,D). Radiographic evaluation revealed that the Insall–Salvati ratio after TKA had a minor change in the PSI-TKA group (*p* = 0.005, Figure 3C), which was 0.05 ± 0.04 in the PSI-TKA group and 0.06 ± 0.05 in the CI-TKA group. Similarly, the mCD ratio after TKA also had a minor change in the PSI-TKA group (*p* < 0.001, Figure 3D), which was 0.06 ± 0.04 in the PSI-TKA group and 0.14 ± 0.08 in the CI-TKA group.

### Changes of ACO and PCO after TKA

**Figure 4:**
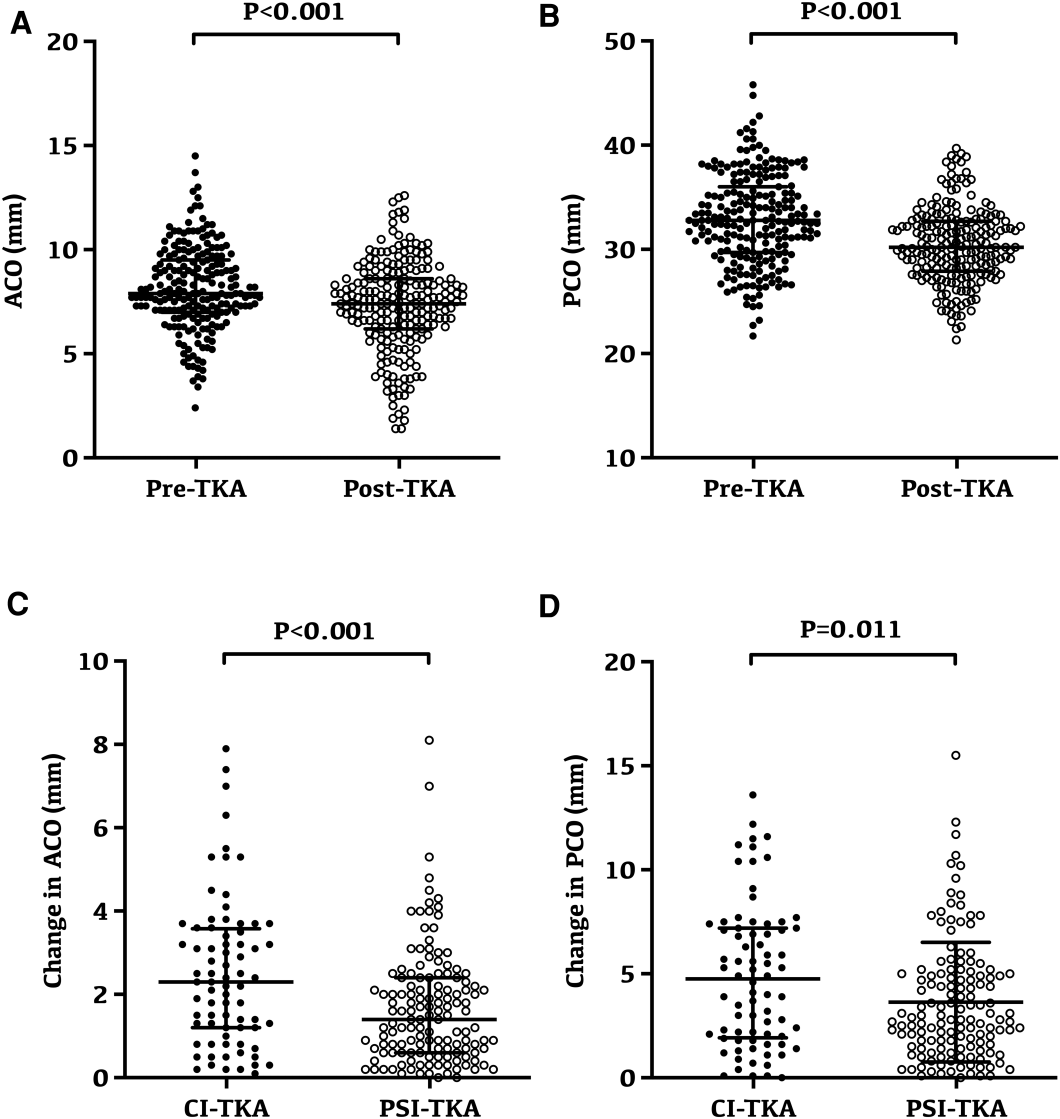
Changes and variability of anterior condylar offset and posterior condylar offset following TKA. (**A**) Preoperative and postoperative anterior condylar offset for all patients; the anterior condylar offset was 8.14 ± 2.01 mm preoperatively and reduced to 7.29 ± 2.21 mm after TKA (*p* < 0.001). (**B**) Preoperative and postoperative posterior condylar offset for all patients; the posterior condylar offset was 32.87 ± 4.40 mm preoperatively and reduced to 30.39 ± 3.60 mm after TKA (*p* < 0.001). (**C**) Anterior condylar offset after TKA had a minor change in the PSI-TKA group (*p* < 0.001). (**D**) Posterior condylar offset after TKA had a minor change in the PSI-TKA group (*p* = 0.011).

Overall, both ACO and PCO were significantly reduced after TKA (Figures 4A,B). The ACO was 8.14 ± 2.01 mm preoperatively and reduced to 7.29 ± 2.21 mm after TKA (*p* < 0.001, Figure 4A). Similarly, the PCO was 32.87 ± 4.40 mm preoperatively and reduced to 30.39 ± 3.60 mm after TKA (*p* < 0.001, Figure 4B).

Radiographic evaluation revealed that the ACO after TKA had a minor change in the PSI-TKA group (*p* < 0.001, Figure 4C), which was 1.67 ± 1.37 mm in the PSI-TKA group and 2.50 ± 1.78 mm in the CI-TKA group. Moreover, the PCO after TKA also had a smaller change in the PSI-TKA group (*p* = 0.011, Figure 4D), which was 3.64 ± 2.88 mm in the PSI-TKA group and 4.89 ± 3.43 in the CI-TKA group.

### Change of PTS after TKA

**Figure 5:**
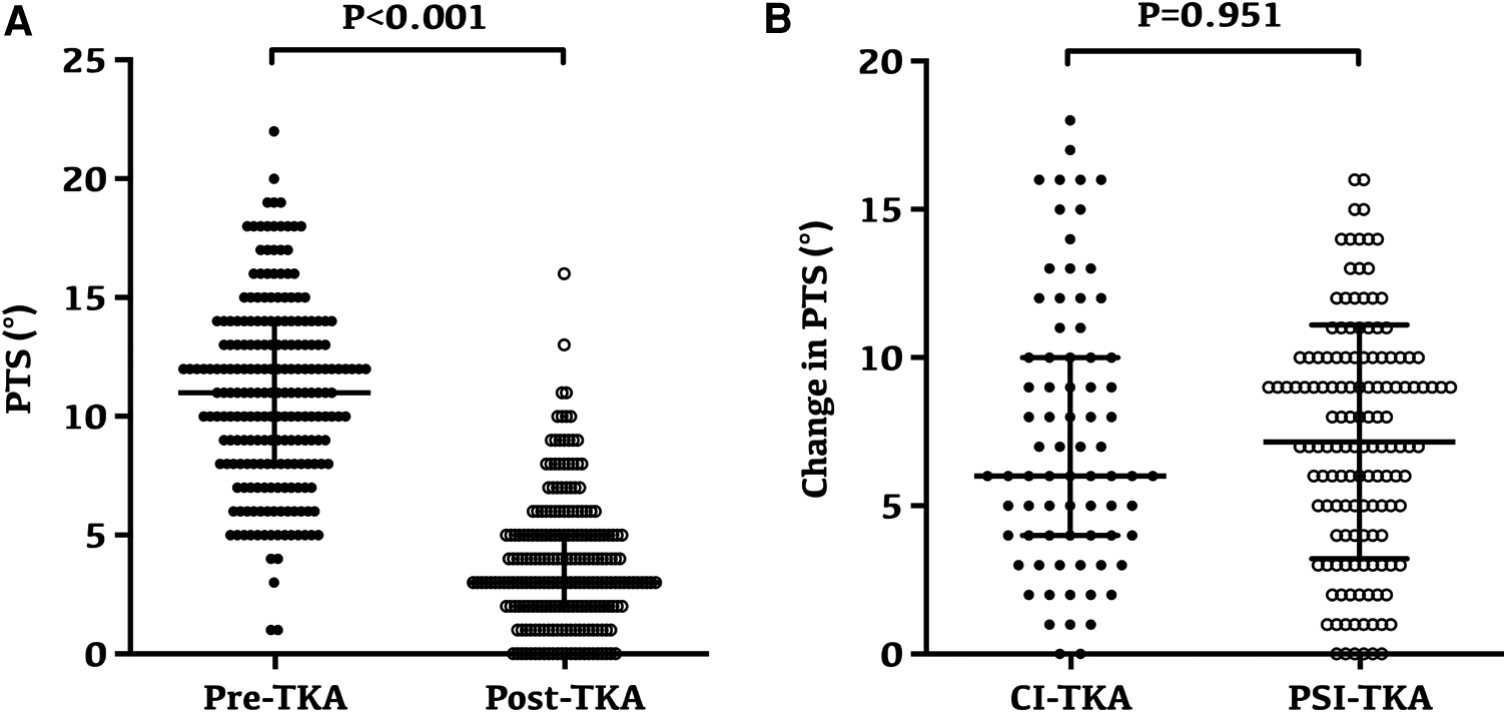
Changes and variability of posterior tibial slope following TKA. (**A**) Preoperative and postoperative posterior tibial slope for all patients; the posterior tibial slope was 10.92° ± 3.88° preoperatively and reduced to 3.78° ± 2.79° after TKA (*p* < 0.001). (**B**) No significant difference in the change of PTS after TKA between the two groups (*p* = 0.951).

Overall, the PTS after TKA was significantly lower than that before the surgery, which was 10.92° ± 3.88° preoperatively and reduced to 3.78° ± 2.79° after TKA (*p* < 0.001, Figure 5A). However, the radiographic evaluation revealed no significant difference in the change of PTS after TKA between the two groups (*p* = 0.951, Figure 5B).

## Discussion

The most important finding of this study was that the variability of patellar height was sufficiently minimized with accurate anterior and posterior femoral condyle osteotomy in the PSI-TKA group; this replied to our second and third study questions. However, although the variability of patellar height was less in the PSI-TKA group, the postoperative Insall–Salvati ratio and mCD ratio declined in both groups; this replied to our first study question. The reduction in the variability of patellar height stemmed from restraining the variation of the ACO and PCO in the PSI-TKA group but had no correlation to the PTS; this also replied to our third study question.

Many previous studies have proved that the maintenance of patella height was very important in TKA. The change of patella height could cause abnormal patella trajectory; cause a series of symptoms related to the patellofemoral joint after TKA; affect the ROM of the knee, postoperative satisfaction, and functional scores; and even require knee revision surgery in severe cases (2–4, 20). In TKA, there is a tendency to vary the patella height due to three main reasons: The first is overstuffing or understuffing in the patellofemoral joint, usually due to the inappropriate size of femoral components, artificially changing the position of femoral components (anterior-posterior position or flexion angle) to prevent notching in the anterior femoral cortex, so that the ACO after TKA is inconsistent with the original size before surgery (21). Second, surgeons have a subjective tendency to overly aggressive distal femoral resection to correct severe preoperative flexion contractures (5). The third is the relatively larger flexion space compared to the extension space when postoperative PCO is decreased or postoperative PTS is increased due to design or intraoperative manipulation issues (3).

3D printed patient-specific instrumentation provides a new solution for achieving neutral alignment in TKA (7). Surgeons can determine the size and position of the implant based on the reconstructed 3D images of full-length lower extremities preoperatively; also, patient-specific cutting blocks can be designed and printed to assist TKA (10). Numerous studies have compared TKA using PSI to TKA performed with CI, proved that PSI could acquire an equivalent or more favorable alignment than CI, such as HKA, alpha, and beta angles (22–24), and improved the accuracy of implant size, positioning, and rotational alignment (25, 26). Whether 3D printed patient-specific instrumentation can reduce the change of patella height while improving the implant accuracy in TKA is still unclear. To the best of our knowledge, none have studied the additional value of 3D printed patient-specific instrumentation for maintaining patella height in TKA.

This study found that the variability of patellar height was sufficiently minimized with accurate anterior and posterior femoral condyle osteotomy when 3D printed patient-specific instrumentation was used. This result suggests that the 3D printed patient-specific instrumentation has the advantage of reducing the change of patellar height after TKA, which is helpful for maintaining patellar height after TKA. However, it is important to note that, even with the 3D printed patient-specific instrumentation, the overall patellar height after TKA was lower than that before the surgery. Therefore, in the preoperative planning and design of TKA, we should not only focus on correcting the alignment of the femoral and tibial components but also pay attention to maintaining the original anatomy of the patients; the latter is very important to maintain the balance of the patient's bone and soft tissue and to get a more natural and original joint feeling after surgery.

ACO is an important factor affecting the stuffing of the patellofemoral joint in TKA. Previous studies showed that femoral components larger or smaller than the anterior condyle resected might lead to the overstuffing or understuffing in the patellofemoral joint and contribute to the alteration in the patellar height (27, 28). This study found a reduced trend in ACO after TKA in both groups. A smaller ACO after TKA might lead to the understuffing in the patellofemoral joint, cause shortening of the patellar tendon and lower patellar height, decrease contact forces in the patellofemoral joint, decrease the moment arm of the knee-extensor mechanism, and ultimately result in a reduction in the moment arm of the knee extensor. Interestingly, this study also found that ACO had a significantly smaller change when 3D printed patient-specific instrumentations were used. It is likely that the reduction in the variability of patellar height might stem from smaller postoperative ACO changes. When 3D printed patient-specific instrumentations were used, based on the patient's preoperative CT data, surgeons could adjust the anteroposterior position of the femoral component during preoperative planning and osteotomy guide design, minimize the ACO changes in TKA, and ultimately reduce its impact on the patellofemoral joint.

PCO is defined as the maximum thickness of the posterior condyle projecting to the tangent of the posterior cortex of the femoral shaft, which can affect knee ROM during flexion. A 1 mm decrease of PCO may reduce knee flexion by 3.3°–6.2° (29). To avoid impingement of the posterior border between the tibial plateau and femur, PCO should be restored to avoid over-resecting the posterior femoral condyle in TKA (30, 31). However, PCO is not restored using standard instrumentation of different manufacturers, impairing the functional improvements after TKA (32). Consistent with previous studies, our study found a trend in over-resection of the posterior femoral condyle in TKA. Fortunately, compared with conventional instrumentation, this study detected that 3D printed patient-specific instrumentations could achieve a significantly smaller change in the PCO. According to previous studies, a smaller PCO can increase the flexion space of the knee joint, which indirectly affects the patella height after TKA (3). It is likely that the smaller change in the PCO with 3D-printed patient-specific instrumentations during TKA might also contribute to minimizing the variability of patellar height after TKA.

PTS is also an important variable during TKA procedures, which is the posterior inclination of the tibial plateau in the sagittal plane (33). An appropriate PTS is a bony factor contributing to anteroposterior (AP) stability and also provides sufficient space to avoid a tight knee in flexion (34). In TKA, an inappropriate cutting angle of the PTS results in polyethylene wear, component loosening, and posterior cruciate ligament (PCL) strain and even affects the patellofemoral joint (3, 35–37). Previous studies reported a significant correlation between PCO and PTS in the medial compartment, probably serving as a sagittal balance between flexion and stability (38, 39). It is also well known that standard TKA alignment techniques and current prosthetic designs of the knee universally ignore the asymmetry of the articular surface and would not reproduce the anatomy in a high percentage of native knees, which may be the reasons for nonphysiologic, paradoxical kinematics after TKA (40, 41). However, the application of 3D printed patient-specific instrumentations during TKA makes it possible to restore the anatomical structures in PCO and PTS of native knees. However, due to the design characteristics of the prosthesis, it is still necessary to appropriately reduce the PTS during preoperative planning and osteotomy guide design to compensate for the PCO reduction caused by matching the balance of the distal condyle and posterior condyle during femoral osteotomy. Consistent with previous studies, our study demonstrated that the PTS and PCO after TKA were significantly lower than those before the surgery in both groups, which served as a sagittal balance between flexion and stability (decreased PCO, decreased PTS). The results replied to our first hypothesis but only partly. The reduction of PCO and PTS may be mainly to balance the flexion and stability of the knee, but the reduction of ACO is one of the main reasons for the change in patellar height after TKA. These also imply that the medial-pivot concept has a great advantage in reconstructing the proper morphology of the posterior part of the knee joint and obtaining a full range of motion in flexion after TKA (39, 42). Furthermore, this study did not detect a significant difference in PTS between the PSI-TKA group and the CI-PSA group. Therefore, it is likely that the reduction in the variability of patellar height in the PSI-TKA group was not due to restraining the change in PTS.

The present study has several limitations: First, our research was a real-world study not a strictly randomized controlled trial. Although surgeons responsible for imaging measurements were blinded to study design and patients' information, some selection bias might still exist. Second, although we overlapped the medial and lateral femoral condyles on sagittal planes at approximately 30° of knee flexion to standardize the lateral radiographs, the measurement errors by a difference in the rotational position in plain radiographs were inevitable. Third, this study compared two different prostheses, although both were posteriorly stabilized knee prostheses, and previous studies did not find differences in the effects of the two prostheses on ACO, PCO, and PTS. However, the impact of the prosthesis on the patella height cannot be ignored.

## Conclusion

This study demonstrated that the variability of patellar height was sufficiently minimized with more accurate anterior and posterior femoral condyle osteotomy when 3D printed patient-specific instrumentation was used. Furthermore, in this study, we also demonstrated a trend in over-resection of the femoral anterior and posterior condyle and a marked reduction in PTS during TKA, which could lead to the change in patellar height and might result in more patellofemoral complications following TKA. These suggest that even with the addition of 3D printed patient-specific instrumentation, the asymmetry of the articular surface would be universally ignored and could not reproduce the anatomy in a high percentage of native knees, which may be the reasons for nonphysiologic, paradoxical kinematics after TKA.

## Data Availability

The raw data supporting the conclusions of this article will be made available by the authors without undue reservation.

## References

[1] Lenhart RL Brandon SC Smith CR Novacheck TF Schwartz MH Thelen DG. Influence of patellar position on the knee extensor mechanism in normal and crouched walking. J Biomech. (2017) 51:1–7. 10.1016/j.jbiomech.2016.11.052 27939752 PMC5204307

[2] Bugelli G Ascione F Cazzella N Franceschetti E Franceschi F Dell'Osso G Pseudo-patella baja: a minor yet frequent complication of total knee arthroplasty. Knee Surg Sports Traumatol Arthrosc. (2018) 26:1831–7. 10.1007/s00167-017-4828-8 29273898

[3] Figgie HE 3rd Goldberg VM Heiple KG Moller HS 3rd Gordon NH. The influence of tibial-patellofemoral location on function of the knee in patients with the posterior stabilized condylar knee prosthesis. J Bone Joint Surg Am. (1986) 68:1035–40. 10.2106/00004623-198668070-00009 3745240

[4] Petersen W Ellermann A Gosele-Koppenburg A Best R Rembitzki IV Bruggemann GP Patellofemoral pain syndrome. Knee Surg Sports Traumatol Arthrosc. (2014) 22:2264–74. 10.1007/s00167-013-2759-6 24221245 PMC4169618

[5] Chonko DJ Lombardi AV Jr. Berend KR. Patella baja and total knee arthroplasty (TKA): etiology, diagnosis, and management. Surg Technol Int. (2004) 12:231–8. PMID: 15455331 15455331

[6] Gromov K Korchi M Thomsen MG Husted H Troelsen A. What is the optimal alignment of the tibial and femoral components in knee arthroplasty? Acta Orthop. (2014) 85:480–7. 10.3109/17453674.2014.940573 25036719 PMC4164865

[7] Camarda L D'Arienzo A Morello S Peri G Valentino B D'Arienzo M. Patient-specific instrumentation for total knee arthroplasty: a literature review. Musculoskelet Surg. (2015) 99:11–8. 10.1007/s12306-014-0339-7 25304253

[8] Chotanaphuti T Wangwittayakul V Khuangsirikul S Foojareonyos T. The accuracy of component alignment in custom cutting blocks compared with conventional total knee arthroplasty instrumentation: prospective control trial. Knee. (2014) 21:185–8. 10.1016/j.knee.2013.08.003 23999209

[9] Hamid KS Matson AP Nwachukwu BU Scott DJ Mather RC 3rd DeOrio JK. Determining the cost-savings threshold and alignment accuracy of patient-specific instrumentation in total ankle replacements. Foot Ankle Int. (2017) 38:49–57. 10.1177/1071100716667505 27649973

[10] Kwon OR Kang KT Son J Suh DS Heo DB Koh YG. Patient-specific instrumentation development in TKA: 1st and 2nd generation designs in comparison with conventional instrumentation. Arch Orthop Trauma Surg. (2017) 137:111–8. 10.1007/s00402-016-2618-2 28005167

[11] Maus U Marques CJ Scheunemann D Lampe F Lazovic D Hommel H No improvement in reducing outliers in coronal axis alignment with patient-specific instrumentation. Knee Surg Sports Traumatol Arthrosc. (2018) 26:2788–96. 10.1007/s00167-017-4741-1 29071356

[12] Insall JN Dorr LD Scott RD Scott WN. Rationale of the Knee Society clinical rating system. Clin Orthop Relat Res. (1989) 248:13–4. PMID: 2805470 2805470

[13] Bellamy N Buchanan WW Goldsmith CH Campbell J Stitt LW. Validation study of WOMAC: a health status instrument for measuring clinically important patient relevant outcomes to antirheumatic drug therapy in patients with osteoarthritis of the hip or knee. J Rheumatol. (1988) 15:1833–40. PMID: 3068365 3068365

[14] Colebatch AN Hart DJ Zhai G Williams FM Spector TD Arden NK. Effective measurement of knee alignment using AP knee radiographs. Knee. (2009) 16:42–5. 10.1016/j.knee.2008.07.007 18790641

[15] Hashemi J Chandrashekar N Gill B Beynnon BD Slauterbeck JR Schutt RC Jr. The geometry of the tibial plateau and its influence on the biomechanics of the tibiofemoral joint. J Bone Joint Surg Am. (2008) 90:2724–34. 10.2106/JBJS.G.01358 19047719 PMC2663332

[16] Insall J Salvati E. Patella position in the normal knee joint. Radiology. (1971) 101:101–4. 10.1148/101.1.101 5111961

[17] Caton JH Prudhon JL Aslanian T Verdier R. Patellar height assessment in total knee arthroplasty: a new method. Int Orthop. (2016) 40:2527–31. 10.1007/s00264-016-3256-6 27503481

[18] Beldman M Breugem SJ van Jonbergen HP. Overstuffing in total knee replacement: no effect on clinical outcomes or anterior knee pain. Int Orthop. (2015) 39:887–91. 10.1007/s00264-014-2548-y 25307257

[19] Hafez MA Hamza H Nabeel A. Hospital-based patient-specific templates for total knee arthroplasty: a proof of concept clinical study. Tech Orthop. (2018) 33:258–63. 10.1097/BTO.0000000000000253 30542227 PMC6250267

[20] Matsuda S Miura H Nagamine R Urabe K Hirata G Iwamoto Y. Effect of femoral and tibial component position on patellar tracking following total knee arthroplasty: 10-year follow-up of Miller-Galante I knees. Am J Knee Surg. (2001) 14:152–6. PMID: 11491425 11491425

[21] Matz J Lanting BA Howard JL. Understanding the patellofemoral joint in total knee arthroplasty. Can J Surg. (2019) 62:57–65. 10.1503/cjs.001617 30693747 PMC6351265

[22] Lee SH Song EK Seon JK Seol YJ Prakash J Lee WG. A comparative study between patient-specific instrumentation and conventional technique in TKA. Orthopedics. (2016) 39:S83–7. 10.3928/01477447-20160509-09 27219736

[23] Sorin G Pasquier G Drumez E Arnould A Migaud H Putman S. Reproducibility of digital measurements of lower-limb deformity on plain radiographs and agreement with CT measurements. Orthop Traumatol Surg Res. (2016) 102:423–8. 10.1016/j.otsr.2016.02.009 27052940

[24] Vaishya R Vijay V Birla VP Agarwal AK. Computerized tomography based “patient specific blocks” improve postoperative mechanical alignment in primary total knee arthroplasty. World J Orthop. (2016) 7:426–33. 10.5312/wjo.v7.i7.426 27458553 PMC4945509

[25] Aman ZS DePhillipo NN Peebles LA Familiari F LaPrade RF Dekker TJ. Improved accuracy of coronal alignment can be attained using 3D printed PSI for knee osteotomies: a systemic review of level III and IV studies. Arthroscopy. (2022) S0749-8063:143–8. 10.1016/j.arthro.2022.02.023 35247513

[26] Sun ML Zhang Y Peng Y Fu DJ Fan HQ He R. Accuracy of a novel 3D-printed patient-specific intramedullary guide to control femoral component rotation in total knee arthroplasty. Orthop Surg. (2020) 12:429–41. 10.1111/os.12619 32087620 PMC7189049

[27] Kandhari VK Desai MM Bava SS Wade RN. Digging deeper into the patello-femoral joint: patello-femoral composite – A new dimension for overstuffing of patello-femoral joint. J Clin Diagn Res. (2017) 11:RC04–7. 10.7860/JCDR/2017/23192.9546 28511465 PMC5427391

[28] Kawahara S Matsuda S Fukagawa S Mitsuyasu H Nakahara H Higaki H Upsizing the femoral component increases patellofemoral contact force in total knee replacement. J Bone Joint Surg Br. (2012) 94:56–61. 10.1302/0301-620X.94B1.27514 22219248

[29] Malviya A Lingard EA Weir DJ Deehan DJ. Predicting range of movement after knee replacement: the importance of posterior condylar offset and tibial slope. Knee Surg Sports Traumatol Arthrosc. (2009) 17:491–8. 10.1007/s00167-008-0712-x 19139846

[30] Arabori M Matsui N Kuroda R Mizuno K Doita M Kurosaka M Posterior condylar offset and flexion in posterior cruciate-retaining and posterior stabilized TKA. J Orthop Sci. (2008) 13:46–50. 10.1007/s00776-007-1191-5 18274855

[31] Bellemans J Banks S Victor J Vandenneucker H Moemans A. Fluoroscopic analysis of the kinematics of deep flexion in total knee arthroplasty. Influence of posterior condylar offset. J Bone Joint Surg Br. (2002) 84:50–3. 10.1302/0301-620X.84B1.0840050 11837832

[32] Wuertele N Beckmann J Meier M Huth J Fitz W. Posterior condylar resections in total knee arthroplasty: current standard instruments do not restore femoral condylar anatomy. Arch Orthop Trauma Surg. (2019) 139:1141–7. 10.1007/s00402-019-03221-8 31209615

[33] Ahmad R Patel A Mandalia V Toms A. Posterior tibial slope: effect on, and interaction with, knee kinematics. JBJS Rev. (2016) 4:e31–6. 10.2106/JBJS.RVW.O.00057 27487427

[34] Giffin JR Vogrin TM Zantop T Woo SL Harner CD. Effects of increasing tibial slope on the biomechanics of the knee. Am J Sports Med. (2004) 32:376–82. 10.1177/0363546503258880 14977661

[35] Hofmann AA Bachus KN Wyatt RW. Effect of the tibial cut on subsidence following total knee arthroplasty. Clin Orthop Relat Res. (1991) 269:63–9. PMID: 1864058 1864058

[36] Singerman R Dean JC Pagan HD Goldberg VM. Decreased posterior tibial slope increases strain in the posterior cruciate ligament following total knee arthroplasty. J Arthroplasty. (1996) 11:99–103. 10.1016/S0883-5403(96)80167-7 8676126

[37] Waelchli B Romero J. Dislocation of the polyethylene inlay due to anterior tibial slope in revision total knee arthroplasty. Knee Surg Sports Traumatol Arthrosc. (2001) 9:296–8. 10.1007/s001670100203 11685361

[38] Bao L Rong S Shi Z Wang J Zhang Y. Measurement of femoral posterior condylar offset and posterior tibial slope in normal knees based on 3D reconstruction. BMC Musculoskelet Disord. (2021) 22:486. 10.1186/s12891-021-04367-6 34044787 PMC8157755

[39] Cinotti G Sessa P Ripani FR Postacchini R Masciangelo R Giannicola G. Correlation between posterior offset of femoral condyles and sagittal slope of the tibial plateau. J Anat. (2012) 221:452–8. 10.1111/j.1469-7580.2012.01563.x 22946518 PMC3482353

[40] Calek AK Hochreiter B Hess S Amsler F Leclerq V Hirschmann MT High inter- and intraindividual differences in medial and lateral posterior tibial slope are not reproduced accurately by conventional TKA alignment techniques. Knee Surg Sports Traumatol Arthrosc. (2022) 30:882–9. 10.1007/s00167-021-06477-z 33547913

[41] Ranawat CS Komistek RD Rodriguez JA Dennis DA Anderle M. In vivo kinematics for fixed and mobile-bearing posterior stabilized knee prostheses. Clin Orthop Relat Res. (2004) 418:184–90. 10.1097/00003086-200401000-00030 15043113

[42] Fitch DA Sedacki K Yang Y. Mid- to long-term outcomes of a medial-pivot system for primary total knee replacement: a systematic review and meta-analysis. Bone Joint Res. (2014) 3:297–304. 10.1302/2046-3758.310.2000290 25325997 PMC4212805

